# Draft Genome Sequence of Halomonas sp. CSM-2, a Moderately Halophilic Bacterium Isolated from a Triassic Salt Mine

**DOI:** 10.1128/MRA.00836-18

**Published:** 2018-07-26

**Authors:** Julianne Megaw, Brendan F. Gilmore

**Affiliations:** a School of Pharmacy, Queen’s University Belfast, Belfast, United Kingdom; University of California, Riverside

## Abstract

Here, we report the draft genome sequence of Halomonas sp. CSM-2. This moderately halophilic bacterium was isolated from a brine sample obtained from a Triassic salt mine.

## Abstract (precis)

Here, we report the draft genome sequence of Halomonas sp. CSM-2.

## ANNOUNCEMENT

The genus Halomonas contains Gram-negative rod-shaped bacteria, first described in 1980 after the isolation of the new genus and species Halomonas elongata from a solar salt facility in the Netherlands (1). Representatives of this genus have since been isolated from diverse saline environments, such as solar salt facilities, intertidal estuaries, the open ocean, and hypersaline lakes. Halomonas species have been described as halotolerant, or slight to moderate halophiles, capable of growth in NaCl concentrations of 0.1 to 32.5% (wt/vol) (2).

Strain CSM-2 was isolated from a sample of brine collected from the Kilroot salt mine, a Triassic halite deposit located in Carrickfergus, Northern Ireland, onto LB agar containing 10% NaCl at 37°C. The strain showed no growth in the absence of salt but grew in the salinity range of 5 to 25% NaCl, with an optimum of 10%, indicating that it is a moderate halophile. Based on 16S sequence similarity, the organism was found to be a member of the genus Halomonas, with the closest neighbor deemed to be the type strain Halomonas janggokensis M24 (99% similarity) (3).

Genomic DNA was extracted from a culture freshly grown in LB broth with 10% NaCl using a GenElute bacterial genomic DNA kit (Sigma-Aldrich, UK), following the protocol for Gram-negative bacteria, yielding 37.2 μg DNA. Whole-genome sequencing was performed by MR DNA (Shallowater, TX), using the Illumina MiSeq platform, on a prepared library with an average size of 739 bp. The resulting 150-bp paired-end reads were assembled *de novo* using the NGen DNA assembly software by DNAStar, Inc. The assembled genome had a total size of 4,131,419 bp in 55 contigs (the largest was 325,242 bp), with an *N*_50_ value of 190,775 bp, an *L*_50_ value of 9, and a GC content of 56.9%. Annotation in Rapid Annotations using Subsystems Technology (RAST) (4) revealed 485 subsystems, 3,877 coding sequences, and 73 RNAs. Several genes were identified that were associated with resistance to heavy metals and toxic compounds (copper, cobalt, zinc, cadmium, mercury, arsenic, and chromium) and antibiotic resistance (fluoroquinolones, β-lactams, and multidrug resistance genes), as well as bacteriocin production and an omega-transaminase, which has since been cloned and expressed in Escherichia coli and shown to be active, with no decrease in activity up to 1.5 M NaCl (5).

### Data availability.

This whole-genome shotgun project has been deposited at DDBJ/ENA/GenBank under the accession number NBYR00000000. The version described in this paper is the first version, NBYR01000000.
